# The Interplay between Insulin Resistance, Inflammation, Oxidative Stress, Base Excision Repair and Metabolic Syndrome in Nonalcoholic Fatty Liver Disease

**DOI:** 10.3390/ijms222011128

**Published:** 2021-10-15

**Authors:** Sylwia Ziolkowska, Agata Binienda, Maciej Jabłkowski, Janusz Szemraj, Piotr Czarny

**Affiliations:** 1 Department of Medical Biochemistry, Medical University of Lodz, 92-215 Lodz, Poland; janusz.szemraj@umed.lodz.pl (J.S.); piotr.czarny@umed.lodz.pl (P.C.); 2 Department of Biochemistry, Faculty of Medicine, Medical University of Lodz, 92-215 Lodz, Poland; agata.binienda@stud.umed.lodz.pl; 3 Department of Infectious and Liver Diseases, Medical University of Lodz, 91-347 Lodz, Poland; maciej.jablkowski@umed.lodz.pl

**Keywords:** nonalcoholic fatty liver disease, insulin resistance, inflammation, DNA repair, oxidative stress, base excision repair, metabolic syndrome

## Abstract

One of the most common chronic liver disorders, affecting mainly people in Western countries, is nonalcoholic fatty liver disease (NAFLD). Unfortunately, its pathophysiological mechanism is not fully understood, and no dedicated treatment is available. Simple steatosis can lead to nonalcoholic steatohepatitis and even to fibrosis, cancer, and cirrhosis of the liver. NAFLD very often occurs in parallel with type 2 diabetes mellitus and in obese people. Furthermore, it is much more likely to develop in patients with metabolic syndrome (MS), whose criteria include abdominal obesity, elevated blood triacylglycerol level, reduced high-density lipoprotein cholesterol level, increased blood pressure, and high fasting glucose. An important phenomenon in MS is also insulin resistance (IR), which is very common in NAFLD. Liver IR and NAFLD development are linked through an interaction between the accumulation of free fatty acids, hepatic inflammation, and increased oxidative stress. The liver is particularly exposed to elevated levels of reactive oxygen species due to a large number of mitochondria in hepatocytes. In these organelles, the main DNA repair pathway is base excision repair (BER). The present article will illustrate how impairment of BER may be related to the development of NAFLD.

## 1. Introduction

One of the most common liver disorders is nonalcoholic fatty liver disease (NAFLD). Approximately 30% of the global population suffer from this condition [1]. Although it is a common ailment, the molecular mechanism responsible for its development remains elusive, and hence the only treatment is based on diets and medications administered to relieve the symptoms. A key problem is that NAFLD does not present any signs throughout the course of its development, and only non-specific symptoms such as abdominal pain or weakness are typically observed in its advanced state [2]. 

NAFLD is mainly associated with type 2 diabetes (T2DM) but is also related to metabolic syndrome (MS) [3]. The criteria for determining MS overlap with the symptoms of NAFLD, such as abdominal obesity, elevated triacylglycerols (TG) level in the blood, decreased high-density lipoprotein (HDL) cholesterol, increased blood pressure, and high fasting glucose. These elevated biochemical parameters are most often associated with an inadequate diet and lifestyle, which can result in liver damage, as well as other diseases such as obesity [4]. 

The main common denominator of NAFLD and MS symptoms is insulin resistance (IR) [5]. It is a major component in the two-hit hypothesis explaining the mechanism of the progression of simple steatosis (NAFL) to more aggressive nonalcoholic steatohepatitis (NASH), in which the increasing level of fibrosis causes cirrhosis and even liver cancer. The first hit is based on the combination of IR with fat accumulation in hepatocytes, while the second hit comprises inflammation, damage to liver cells, and consequent fibrosis. However, the two-hit hypothesis is not a sufficient explanation of the progression mechanism, as genetic and epigenetic factors may also be involved, as well as the intestinal microflora [6].

The key aspects in the development of NAFLD are the presence of liver inflammation, increased oxidative stress, and fat accumulation; these are linked through IR. For example, oxidative stress is known to enhance inflammation and play an important role in the IR process [7]. High levels of reactive oxygen species (ROS) are formed in mitochondria due to the functioning of the electron transport chain (ETC). This elevates the level of oxidative stress near the mitochondrial DNA (mtDNA), thus increasing the likelihood of DNA damage [8]. If the DNA repair pathways become impaired, this would lead to more stress and, consequently, to an increased IR. The main DNA repair system in mitochondria is base excision repair (BER): a complex process whose activity involves a range of proteins, and failure to perform even one of its steps results in a reduction in the efficiency of the process [9].

The exact molecular mechanisms of the development and progression of NAFLD remain elusive, particularly the impact of BER on IR. The present article provides an overview of the current state of knowledge regarding the relationship between IR, inflammation, oxidative stress, mitochondrial dysfunction, BER, and metabolic syndrome in NAFLD. We suspect that the DNA repair pathway may play an important role in the pathomechanism of NAFLD, and it is closely associated with the molecular background of the disease. A search was made of PubMed to identify papers focusing on the molecular background of NAFLD in animal models and human subjects. The following keywords were applied: NAFLD, fatty liver, nonalcoholic fatty liver, nonalcoholic steatohepatitis, Kupffer cells, insulin resistance, metabolic syndrome, T2DM, obesity, liver inflammation, mitochondrial dysfunction, oxidative stress, high-fat diet, methionine and choline deficient diet, fat accumulation, free fatty acids, BER, and DNA repair.

## 2. Development of NAFLD

The development of NAFLD is influenced by various factors, which are also responsible for its progression and conversion to NASH, characterized by chronic inflammation in the liver. The most important factors of NAFLD have increased lipolysis of triglycerides (TG) and the release of free fatty acids (FFA), which are taken up by the liver [1]. Elevated FFA levels also indirectly contribute to the development of inflammation and are known to exacerbate IR. In addition, oxidative stress may also be a critical factor in the development of inflammation and IR. These disturbances are reminiscent of a metabolic disorder. Due to this fact, NAFLD is called the hepatic manifestation of a MS [10].

### 2.1. Epidemiology and Etiology

**Figure 1:**
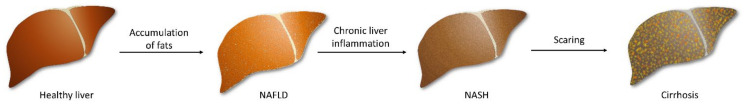
The development of fatty liver beginning with the accumulation of fats in primarily healthy hepatocytes. Prolonged inflammation in the fatty liver leads to steatosis and fibrosis of tissue. The following scaring of the liver triggers irreversible damage, and cirrhosis occurs.

NAFLD is characterized by lipid accumulation in hepatocytes and abnormal liver enzyme levels. Steatosis is diagnosed when at least 5% of liver cells contain excess fat [11], particularly in people who do not drink alcohol or limit their consumption to 30 g of alcohol (men) and 20 g (women) per day [12]. Furthermore, about one-fifth of NAFLD patients will develop NASH as a result of chronic liver inflammation [13], characterized by hepatocellular injury and the characteristic ballooning of hepatocytes [14]. Small lesions could easily be regenerated by the liver, but prolonged inflammation causes fibrosis, resulting in scarring of the liver, which can lead to cirrhosis and liver failure. Unfortunately, progressive lesions are also able to trigger hepatocellular carcinoma (Figure 1) [15].

NAFLD is more common in older people [16], and men tend to demonstrate greater frequency and severity than women. However, the incidence increases among postmenopausal women [2]. Moreover, the condition is the most widespread in people living in Western countries and is more prevalent in people of Asian, Hispanic, or Native American ancestry than in Eastern Europeans or Africans [17]. It is speculated that this might be associated with the diet of various populations.

As mentioned above, NAFLD does not show noticeable symptoms for a long time, and these become visible only when the liver is significantly enlarged; furthermore, many of these symptoms are non-specific, such as abdominal discomfort, pain, or fatigue [18]. NAFLD is often diagnosed during laboratory tests when increased levels of the liver enzymes alanine aminotransferase (ALT) and gamma-glutamyl transpeptidase (GGTP) are present. Furthermore, the diagnostic picture of the disease resembles that of metabolic syndrome (MS), i.e., low level of HDL cholesterol, increased level of TG in the blood, hypertension, hyperglycemia, and IR [19]. However, the gold standard of NAFLD diagnosis is liver biopsy. Nevertheless, the other effective methods should be searched and applied, as the surgery is invasive and may trigger complications [20]. 

NAFLD is closely associated with MS and hence is often referred to as metabolic-associated fatty liver disease (MAFLD) [21]. This relationship is also reflected by the similarity of the conditions that occur in both disorders. However, equally important is the fact that NAFLD very often coexists with T2DM [22]. The key common denominator linking NAFLD and T2DM is IR. However, apart from MS and T2DM, other risk factors are obesity (playing an important role in the MS), physical inactivity, and a Western diet rich in fats and carbohydrates, eaten irregularly and too abundantly. The first factor contributes the most to the disease. Obese people demonstrate excess accumulation of fats in adipocytes, which is the essence of steatosis, and even up to 74% of obese individuals have fatty livers. Moreover, obesity is also associated with NAFLD risk factors such as lack of exercise and unhealthy nutrition. A sedentary lifestyle along with an inadequate high-calorie diet, including high fructose and saturated fat intake, can trigger the development of fatty liver [2].

Studies indicate that NAFLD also has a genetic basis. The first gene found to be related to NAFLD was patatin-like phospholipase domain-containing 3 (*PNPLA3)*. It was shown that the rs738409 (c.444C>G) missense variant I148M in this gene, i.e., the replacement of isoleucine with methionine, occurring in the catalytic patatin domain (position 148), a highly-conserved location in vertebrates, was strongly associated with hepatic inflammation and increased liver fat levels [23]. Patatin catalyzes the cleavage of fatty acids from membrane lipids [24]. *PNPLA3* encodes triacylglycerol lipase, also known as adiponutrin, belonging to the patatin-like family. The function of this molecule is not well understood, but it is related to the metabolism of TG in adipocytes. The I148M variant present in NAFLD reduces lipolytic activity against TG, possibly due to limited substrate availability [25]. This leads to decreased lipolysis of fats, and their increased production, in the liver [26]. The I148M variant is also associated with an increased likelihood of hepatocellular carcinoma occurrence [27]. Recent studies have also found other genes to influence NAFLD, e.g., *TM6SF2, GCKR*, or *HSD17B13*. Their products are involved in the synthesis of cholesterol, lipogenesis by regulating the influx of glucose into hepatocytes, and modulation of serum liver enzyme levels, respectively [28]. Moreover, the E167K variant (Glu167Lys) in *TM6SF2* enhances hepatic steatosis and the progression of fibrosis [27]. Increasingly, new research studies indicate a link between NAFLD and the products of various genes.

### 2.2. FFA Accumulation

A Western diet, rich in high-fat and high-sucrose products, may lead to increased FFA accumulation and, in consequence, cause hepatic disorders such as NAFLD [29,30]. Accordingly, a case-control study showed that patients suffering from NAFLD had significantly higher serum FFA profiles compared to healthy controls [31]. There are several possible mechanisms of fatty accumulation in NAFLD, including (i) elevated *de novo* synthesis of fatty acids in hepatocytes, (ii) β-oxidation of fatty acids, (iii) excess dietary fat and carbohydrate intake, and (iv) retention of lipids due to impaired hepatocyte apolipoprotein [30]. 

Lipogenesis enables the liver to synthesizes new fatty acids from non-lipid substrates, like sucrose and glucose. Briefly, carbohydrates are converted to acetyl-CoA, which is a precursor for new fatty acids, which subsequently can be esterified and stored as TG. Acetyl-CoA carboxylase (ACC) and fatty acid synthase (FASN) are the main enzymes involved in this pathway. Patients suffering from NAFLD were found to have higher non-esterified fatty acid (NEFA) concentrations but normal NEFA turnover rates. Moreover, an increase in the contribution of hepatic lipogenesis to TG secretion was observed in patient plasma [32]. Additionally, patients with NAFLD have a higher synthesis of fatty acids as compared to healthy controls [33]. 

*De novo* lipogenesis is mainly regulated by the sterol regulatory element-binding transcription factor 1c (SREBP1c) and the carbohydrate response element-binding protein (ChREBP) [34,35,36]. SREBP1c is activated by insulin and induces the expression of genes involved in glucose utilization, while ChREBP, which is activated by glucose, is mostly expressed in active sites of *de novo* lipogenesis, such as hepatocytes and adipocytes [37]. In vivo studies indicate that SREBP1c knockout mice have decreased expression of lipogenic enzymes, whereas transgenic mice overexpressing SREBP1c have higher hepatic TG levels and insulin levels, indicating IR [38,39]. Interestingly, patients with NAFLD have been found to demonstrate elevated SREBP1 expression [40]. Furthermore, mice with ChREBP knockout have shown fructose, glucose as well as sucrose intolerance. Such fructose intolerance can be associated with the reduction in fructokinase and triose kinase expression [41]. Moreover, a connection has been noted between sucrose intolerance and decreased expression of intestinal sucrose-isomaltase (SI), the glucose transporters 5 (GLUT5) and 2 (GLUT2), as well ketohexokinase (Khk) [42]. Furthermore, *de novo* lipogenesis, and as a result, NAFLD are frequently associated with the IR state, like obesity and type 2 diabetes [43,44]. 

Obese subjects demonstrate an increased ratio of hepatic lipogenesis to the circulating TG pool. Furthermore, energy restriction in obese patients was found to decrease plasma insulin and leptin levels and inhibit normalized hepatic lipogenesis [43]. The increase in hepatic lipogenesis is responsible for the phenotype of IR in the lipid-deficient (ob/ob) mice. Liver-specific inhibition of ChREBP in ob/ob mice decreased lipogenic rates, which was correlated with reduction in hepatic levels of TG and NEFA, and, as a consequence, improved hepatic steatosis [45]. Liver biopsies from patients with NASH demonstrated increased ChREBP expression, where steatosis was greater than 50% and decreased expression in the presence of severe IR [46]. Moreover, mice overexpressing ChREBP fed standard diet remained insulin sensitive, despite increased expression of lipogenesis genes, whereas mice overexpressing ChREBP fed a high-fat diet presented normal insulin levels and improved glucose tolerance as compared to controls, despite having greater hepatic steatosis [46]. Furthermore, mice with liver-specific deletion of acetyl-CoA carboxylase isoform 1 (ACC1) had 40%–70% less TG accumulation; however, after receiving a fat-free diet, the levels of lipogenic enzymes, including FASN, were upregulated [47]. To summarize, many studies indicate that elevated *de novo* lipogenesis is a major mechanism involved in NAFLD pathophysiology. 

Another mechanism of fatty acid accumulation involves β-oxidation. Fatty acid oxidation (FAO) is mainly regulated by peroxisome proliferator-activated receptor γ (PPARγ) and reduces intrahepatic fat levels by utilizing lipids as an energy source [48]. Mice with a high-fat and methionine- and choline-deficient (MCD) diet develop steatohepatitis, which is histologically similar to human metabolic steatohepatitis. Administration of the PPARγ agonist Wy-14,643 significantly decreased the levels of ALT and hepatic lipoperoxides, and improved steatohepatitis [49]. Furthermore, PPARγ knockout mice fed the MCD diet developed more severe steatohepatitis than wild-type (WT) mice and were unaffected by Wy-14,643. The results also indicate that activation of PPARγ correlates with hepatic lipid turnover [50], suggesting that MCD diet-induced fibrosing steatohepatitis can be reversed by PPARγ agonist treatment. Additionally, pharmacological PPARγ activation improves the metabolic milieu and steatosis; it also suppresses NF-κB and c-Jun *N*-terminal kinase (JNK) activation, neutrophil, and F4/80 macrophage recruitment in diabetes-related NASH [51]. PPARα also plays an important role in insulin sensitivity in liver disorders. Three selective PPARα agonists reduced IR without having harmful effects on body weight and adipose tissue mass in animal models of high-fat diet-induced and genetic IR [52]. Additionally, PPARα-null and apoE-null mice fed a high-fat diet had increased suppression of endogenous glucose production in hyperinsulinemic clamp experiments, reflecting less IR in the absence of PPARα [53]. 

Finally, excess dietary fat and carbohydrate intake also take part in the NAFLD pathophysiology [54,55]. Many studies confirmed that a long-term high-fat diet promotes the development of NAFLD [56,57,58]. Patients with NAFLD consume less *n*-3 polyunsaturated fatty acids (PUFAs) and a higher *n*-6/*n*-3 PUFA ratio than healthy controls [59,60]. It is important to note that *n*-3 PUFAs have an anti-inflammatory effect, regulate hepatic lipid consumption, and improve insulin sensitivity [61], while *n*-6 PUFAs are considered pro-inflammatory molecules and contribute to hepatic steatosis [62]. Furthermore, fructose, which is the main component in sucrose and high fructose corn syrup, is a major mediator of NAFLD [63,64]. Two meta-analyses have identified a strong association between sugar-sweetened beverage consumption and increased risk of NAFLD [65,66]. Even a short-term carbohydrate overfeeding had a significant lipogenic effect on the liver [67]. Another study showed that fructose participates in both increased lipogenesis and impaired fat oxidation [63]. Moreover, a linkage has been found between the occurrence of fatty liver and the metabolism of fructose by fructokinase C, resulting in ATP consumption, nucleotide turnover, and the generation of uric acid that mediates fat accumulation [63]. In conclusion, there is strong evidence suggesting that the limitation of fat and sugar from daily diet may have a positive effect on reducing hepatic fat accumulation.

### 2.3. Inflammation 

Obesity, the leading cause of NAFLD, is characterized by increased lipid accumulation in the adipose and liver tissues. The resulting elevated FFA levels are responsible for lipotoxicity and dysfunction in adipose tissue [1]. Disturbances in lipid metabolism, consequent IR, and the presence of gut-derived endotoxins contribute to the production and release of proinflammatory TNF-α, IL-1b, and IL-6 [68]. These cytokines inhibit the signaling of insulin receptors and lower insulin sensitivity in the liver, which leads to the induction of steatosis and fibrosis [69].

In the liver, about 10% of all hepatic cells are Kupffer cells, which are macrophages characterized by increased production of proinflammatory cytokines [70]. They mediate the activation of T cells and the regulation of hepatocyte apoptosis [71]. Due to their direct contribution to inducing inflammation, they play an important role in the development of NASH. Kupffer cells appear as proinflammatory M1 cells and anti-inflammatory M2 cells. The former promotes liver steatosis, fibrogenesis, and elevation of hepatic lipid accumulation, while the latter are resident macrophages of the liver and are responsible for activating M1 apoptosis in a caspase-3-dependent manner [72,73].

Under the influence of proinflammatory resident factors, i.e., interferon-gamma (IFNγ) and ligands of toll-like receptor (TLR), M2 macrophages undergo classical activation to M1 [74]. This follows the production of inflammatory cytokines, including TNF-α and chemokines [74,75]. Chemokines, as a family of cytokines activating leukocyte chemotaxis, contribute to the development of inflammation and secondarily IR. The attachment of the chemokine ligand 2 (CCL-2) to the chemokine receptor 2 (CCR2) results in the migration of the bone marrow-derived macrophages to the liver or adipose tissue [76]. CCL-2 expression is elevated in the adipose tissues of obese patients [77]. Other chemokines important in liver steatosis, RENTES/CCL5, CXCL8, CXCL9, and CXCL10, are significantly elevated in the case of NASH. Additionally, they are involved in cases of increased hepatic fat accumulation, which results in dysregulation of the lipid metabolism [78,79,80].

To relieve inflammation, M1 is stimulated by Il-4 and IL-13 to induce M2 activation [74]. M2 neutralizes proinflammatory cells by apoptosis. This process is critical to the attenuation of the inflammatory response present in NAFLD. The M1/M2 balance is important for regulating inflammation in the liver [73]; however, the M1/M2 ratio is increased during NAFLD progression [81].

Another transcription factor responsible for inducing inflammation is NF-κB. In the liver of the animal models of NAFLD and NASH patients, the NF-κB signaling pathway is continuously active, and its IKK2 subunit is upregulated [82,83]. This upregulation results in the occurrence of chronic inflammation and IR and promotes the development of hepatocellular carcinoma [6]. 

### 2.4. ER Stress

Hepatocytes are rich in the endoplasmic reticulum (ER) [84], which plays a crucial role in protein and lipid biosynthesis. Any disturbances in its functioning may lead to the occurrence of ER stress. This kind of stress stimulates the signaling pathways that induce lipotoxicity, inflammation, and apoptosis [85]. Presumably, as ER stress appears to have an influence on alterations in lipogenesis, it may also contribute to the occurrence of IR. Thus, one can assume that the presence of ER stress has an impact on NAFLD and NASH.

An important process relevant to NAFLD is the unfolded protein response (UPR) [86], i.e., the physiological response of the cell to inappropriate protein maturation, folding, or transfer, caused by a high-fat diet, among others [87]. The aim of the UPR is to increase protein folding and thus stabilize the functioning of the cell. It also contributes to a reduction in protein synthesis, increased degradation of unfolded ones, and upregulation of chaperones, which are responsible for the folding of proteins [88,89]. Moreover, it is responsible for the phosphorylation of Nrf2, by which it activates the transcription of antioxidant enzymes to lower the ROS level [90,91]. However, prolonged UPR activity has a negative effect on energy metabolism and results in the induction of proinflammatory and proapoptotic pathways [12]. Moreover, UPR is also responsible for the induction of ER stress due to the initiation of UPR signal proteins: activating transcription factor 6 (ATF6), pancreatic ER kinase (PKR)-like ER kinase (PERK), and inositol-requiring enzyme 1 (IRE1) [92]. PERK activation blocks the synthesis of proteins, whereas ATF6, together with IRE1, upregulates the expression of ER chaperones genes. IRE1 promotes the mRNA splicing of X box-binding protein 1 (XBP1), and the spliced variant of XBP1 and ATF6 acts as transcription factors. In hepatocytes, XBP1 regulates lipogenesis in the liver and contributes to VLDL. In NAFLD patients, the level of XBP1 is elevated. Mice fed with fructose and with depletion of XBP1 have reduced hepatic lipid accumulation and IR due to JNK signaling activation resulting from IRE1 interactions. XBP1 level is also elevated in patients with T2DM [84].

Recent studies confirm the association between ER stress and IR. ER stress is characterized by the activation of JNK and NF-κB, which play important roles in the inflammation process and IR. JNK induces the phosphorylation of serine (Ser307) in the insulin receptor substrate 1 (IRS1), which impairs insulin signaling [93,94]. In addition, ER stress activates the tribbles 3 (TRB3) protein, by which the Akt/PKB signaling pathway is inhibited, and then the insulin-stimulated glucose uptake is reduced, which may lead to IR [95].

ER stress has been detected in animal models of obesity and animals fed with MCD diet [85,96,97]. Moreover, rapid onset and progression of steatohepatitis were observed in animal models with Nrf2 (factor promoting antioxidant effect) deletion and in those fed with MCD or high-fat diets [84]. Furthermore, increased UPR component activity was noticed in the liver of NAFLD patients [98]. Clearly, ER stress plays an important role in inflammation, insulin signaling, and lipid homeostasis, all of which are crucial in the NAFLD pathogenesis. 

### 2.5. Oxidative Stress

Oxidative stress is one of the main factors associated with obesity and related disorders, such as cardiovascular diseases, T2DM, MS, as well as cancer, and neurodegenerative disorders [99,100]. Oxidative stress arises from the imbalance of ROS generation and neutralization with antioxidants [101]. ROS such as superoxide, hydroxyl radicals, and hydrogen peroxide are physiologically produced in low concentrations by peroxisomes and mitochondria [102,103] and are involved in signaling pathways as factors controlling transcription and the cell cycle [102,104]. However, at higher concentrations, ROS cause DNA damage, alterations in gene expression, chromosomal instability, and lipid oxidation [101]. Oxidative stress is also associated with the mechanism of insulin formation. Recent studies confirm the presence of oxidative stress biomarkers, i.e., malondialdehyde, 7,8-dihydro-8-oxoguanine (8-oxo-dG), or other markers of oxidative damage in cases of IR [105]. Additionally, the presence of elevated oxidative stress is also directly involved in the development and progression of NAFLD [101]. It contributes to impaired mitochondrial, peroxisomal FFA oxidation and altered cytokine release, and consequently results in increased lipid peroxidation and liver inflammation, leading to the formation of stellate cells and fibrogenesis [106]. 

**Figure 2:**
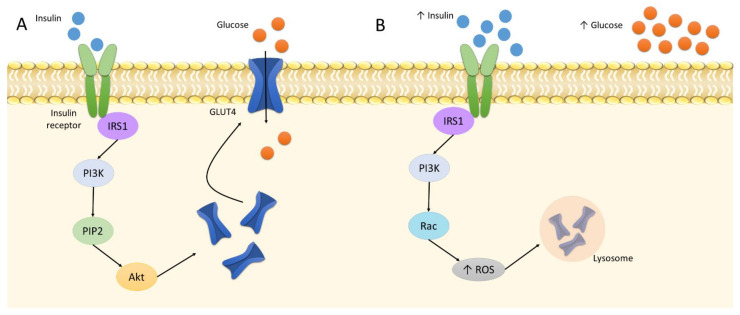
The insulin signaling: A: normal insulin level; B: increased insulin level. Abbreviations: Akt: protein kinase B; IRS1: insulin receptor substrate 1; PI3K: phosphoinositide 3-kinase; PIP2: phosphatidylinositol biphosphate; ROS: reactive oxygen species.

Oxidative stress is related to the etiology of IR. In its physiological state, insulin binds to the insulin receptor to phosphorylate IRS1. This triggers the phosphorylation of PI3 kinases and subsequent PIP2. The cascade activates the Akt pathway [107]. It is followed by the translocation of GLUT4 to the plasma membrane in tissues sensitive to insulin, i.e., adipocytes and skeletal muscles. It increases the cellular absorption of glucose and reduces its concentration in the blood [108]. Insulin elevates GLUT4 expression to increase glucose uptake; however, at increased insulin levels, GLUT4 is downregulated. Four-hour treatment of 1 nM insulin on 3T3-L1 adipocytes was found to decrease the protein level of GLUT4 by approximately 20% [109]. Following this downregulation and the buildup of glucose in the bloodstream, the pancreas produces more insulin due to the positive feedback loop; as a result, the cells become insulin resistant, i.e., insensitive to insulin. In such cases, GLUT4 demonstrates continually lower expression, which results in hyperinsulinemia, hyperglycemia, and increased oxidative stress, leading directly to inflammation [7,109]. In addition, a higher insulin level also alters the action of PI3, which phosphorylates Rac instead of PIP2, and, thus, activates NADPH oxidase 4, producing large amounts of ROS [110]. This involves the activation of the retromer, i.e., a group of proteins engaged in endosomal transport; as a result, GLUT4 is transported to lysosomes for degradation and cannot perform its function (Figure 2) [7].

When the diet is full of glucose-rich meals, the mitochondria produce more ATP. Due to the rise in their activity, the level of ROS also increases [111]. To repair the associated damage, the mitochondrial response stimulates the NF-κB and JNK pathways, both of which influence the pathophysiology of NAFLD [112]. 

### 2.6. Mitochondrial Dysfunction

Mitochondria are the main locations for energy storage and fatty acid metabolism in cells [108]. They maintain the energy homeostasis of the cell, i.e., β-oxidation, ETC, as well as the production of ATP and ROS [113]. Thus, it is not surprising that mitochondria play a significant role in diseases associated with metabolic pathways. More than two decades ago, a relationship between disturbed mitochondrial function and NAFLD, i.e., ETC and beta-oxidation dysfunction, was confirmed [114,115]. Now, it is obvious that mitochondrial dysfunction is associated with insufficient oxidation of fatty acids, leading to elevated ROS levels and, consequently, increased oxidative stress [116]. Free radicals produced in mitochondria stimulate the activity of signaling pathways capable of inducing necroinflammation in liver cells, as well as mitochondrial damage [117]. Thus, mitochondrial dysfunction contributes to the accumulation of FFA in hepatocytes and the development of IR and NAFLD, especially in the case of a fat-rich diet intake [118].

Mitochondrial dysfunction can be triggered by disturbances in the functioning of various families of proteins. One of these families is sirtuins, which are a group of NAD^+^-dependent deacetylases [119]. Sirtuin 1 (SIRT1) mediates the regulation of oxidative stress by upregulating antioxidant enzymes [120], while sirtuin 3 (SIRT3) enhances the level of fatty acid oxidation by stimulating long-chain acyl-CoA dehydrogenases. SIRT3 level was found to be reduced in animals with fatty livers [121]. Additionally, since sirtuins act with NAD^+^, its depletion may result in mitochondrial dysfunction and an increase in FFA level in hepatocytes [122].

Slc25a1, a citrate carrier accessing the inner mitochondrial membrane, which is associated with FFA metabolic processes and glycolytic pathways, is also involved in the proper functioning of mitochondria. The inhibition of Slc25a1 decreases steatosis, protects against steatohepatitis, and reduces the inflammation in adipose tissue [123]. Another mitochondrial transporting molecule, carnitine, is responsible for transferring long-chain fatty acids from cytoplasm to matrix. It inhibits oxidative stress, enhances β-oxidation, and reduces IR. The supplementation of carnitine improves the AST, ALT, TG, and HOMA-IR (indicator of IR) parameters in NAFLD patients [124].

In the case of NAFLD, the main challenge faced by the mitochondria is managing a large amount of fat flowing into the hepatocytes. These organelles are responsible for the transformation of lipotoxic FFA into intracellular TG stores, which limits the increase in oxidative stress [125]. Mice fed an MCD diet with depletion of diacylglycerol O-acyltransferase 2 (DGAT2), a protein involved in the final step of FFA to TG conversion, demonstrate elevated levels of markers of lipid oxidation stress, necroinflammation, and fibrosis [126]. In addition, attenuated β-oxidation leads to the accumulation of lipotoxic intermediates, which also causes inflammation and alters insulin signaling [127].

Recent studies have identified several mechanisms that link mitochondrial dysfunction with obesity and IR [128]. Insulin is important to the mitochondria for two key reasons: firstly, insulin maintains an appropriate NAD^+^/NADH ratio in them, and secondly, free radicals derived from mitochondria modulate insulin sensitivity, while the ROS excess disrupts insulin signaling and induces IR [129].

Moreover, mutations in mtDNA are associated with MS. For instance, a mutation in the *tRNA(Leu)(UUR)* gene causes impairment of insulin secretion from pancreatic β cells [130]. Cytochrome P4502E1 (CYP2E1), which is the source of ROS, is also involved in the metabolism of fatty acids. Its level is increased in the animal models of NASH and NASH patients [131,132]. In addition, some polymorphisms found in CYP2E1 are associated with the occurrence of NASH in obese T2DM patients [133].

Due to the fact that mitochondria produce ROS, antioxidant activity is required. An animal model of NASH was found to demonstrate decreased glutathione peroxidase (GPx) activity, probably caused by the depletion of glutathione and its poor transport to the mitochondrial matrix [134]. Moreover, the occurrence of the C47T polymorphism in the gene encoding superoxide dismutase 2 (SOD2) not only lowers its activity and contributes to an increase in ROS levels but also increases the likelihood of NASH development and advanced fibrosis in NAFLD [135]. These findings demonstrate a clear link between mitochondrial dysfunction and fatty liver disease.

## 3. The Interplay between NAFLD, MS, IR, and DNA Repair

There are many features in the development of NAFLD and MS that play crucial roles in both diseases. For example, both are characterized by altered insulin signaling and insulin sensitivity of cells. In this chapter, we present studies that confirm the link not only between IR, MS, and its associated obesity and T2DM but also the role of DNA repair in the development of IR. This body of evidence highlights the possible relationship between DNA repair, especially BER, and NAFLD itself.

### 3.1. Common Features of NAFLD and MS

Metabolic syndrome (MS) is a cluster of features that emerges from disturbances in the metabolism of fats, sugars, amino acids, or iron. MS is a significant factor that increases the risk of T2DM, cardiovascular diseases, or chronic kidney disorders [136]. To diagnose MS, at least three out of the following five criteria should be confirmed: abdominal obesity, elevated TG level, reduced HDL, hypertension, and disturbances in fasting glucose [137]. It is estimated that over 23% of the general population suffers from MS, and 10% of these patients demonstrate the presence of all five criteria [1].

The key factors in the development of MS are IR and hyperinsulinemia [138]. These features are associated with obesity, which also appears to be a fundamental problem in this condition. This is due to the presence of an increased level of very-low-density lipoproteins (VLDL), functioning as TG carriers, which mediates the hypertriglyceridemia [139]. In addition, elevated FFA flux to hepatic and other tissues may be observed in MS, which also results in an increase in TG levels, and thus contributes to the development of IR [140]. Additionally, fat accumulation in visceral adipose tissue stimulates the production of adipocytokines, e.g., TNF-α and IL-6; this not only leads to inflammation but can also induce hypertension [141]. Hypertension may be caused due to hyperinsulinemia resulting from increased intake of fats. In such cases, insulin stimulates endothelin-1 to increase the proliferation of smooth muscle cells, leading to vasoconstriction [142]. Thus, it is not surprising that due to the IR, the vasodilation of the blood vessels in the liver can be observed. In fatty livers, there is an increased diameter of the portal vein along with a decreased flow velocity. Moreover, the diameter of the portal vein is positively correlated with the severity of NAFLD [143].

The above processes immediately bring to mind the mechanisms occurring in NAFLD, where insulin signaling and disturbances in lipid metabolism are also important factors in the development of the disease [144]. Moreover, 90% of cases of nonalcoholic fatty livers are accompanied by the presence of at least one diagnostic criterium of MS, while 33% have all [1]. Interestingly, fatty livers are much more common in people with MS than in ones without this disorder [145]. A study based on 4401 subjects confirms that the presence of MS increases the likelihood of NAFLD onset almost 10-fold and significantly reduces the chances of disease regression [146]. This suggests that MS is a risk factor for NAFLD.

Due to the numerous similarities and relationships between NAFLD and MS, in 2020, a new concept of the disease, called MAFLD, was proposed [147]. However, the criteria for the diagnosis of this disease are different, as they do not exclude the consumption of alcohol or chronic liver diseases [148]. Moreover, the condition requires the presence of metabolic disorders [149]. Thus, people with MAFLD tend to be older and have a higher body-mass index (BMI), blood pressure, and levels of lipids and hepatic enzymes than NAFLD patients [21].

Despite the strong association of NAFLD with obesity, the disease also occurs in non-obese patients; however, it is less prevalent. Interestingly, it is more common for lean patients to demonstrate metabolic problems [10,150]. Even in non-obese individuals, a relationship can be found between NAFLD and visceral fat tissue: a greater thickness of the visceral fat is related to a greater incidence of MS and severity of NAFLD [151]. This could indicate that visceral fat is the link between these two diseases.

In both cases, the disorder may be caused by inadequate nutrition and lack of physical activity. This promotes an increase in fat accumulation, which correlates positively with hepatic IR, leading to enhanced lipolysis and a rise in circulating FFA levels [125,152]. However, the problem with IR is not restricted to hepatic cells but is also found in other tissues. It has been shown that in human skeletal muscle cells with IR, glucose, stored in muscle as glycogen, is directed to the *de novo* lipogenesis pathway in the liver [153,154]. This may result in the occurrence of NAFLD. Nevertheless, peripheral IR alters the lipid metabolism, leading to the development of liver steatosis. Moreover, the elevated level of lipolysis in white adipose tissue indirectly contributes to the accumulation of lipids in the other tissues, facilitating the formation of hepatic IR [155]. However, both visceral or peripheral fat content induces IR in hepatocytes [10].

The prevalence of MS and NAFLD increases with age. The reason is that fat moves from subcutaneous to visceral adipose tissue with age [10,156,157]. This results in the production of pro-inflammatory cytokines; the level of production increases in adipose tissue with higher fat content, resulting in higher NF-κB activity [158,159,160]. Indeed, inflammation is an essential aspect of the development of both diseases. MS has elevated levels of TNF-α, IL-6, and c-reactive protein (CRP) [161]. In addition, FFA has an indirect impact on toll-like receptor 4 (TLR4), which triggers the NF-κB signaling pathway [162]. TLR4 knockout mice are protected from inflammation caused by IR and lipid influx [162,163]. In humans with T2DM, the observed increase in TLR4 expression in skeletal muscle correlates positively with IR [164,165].

Recent studies indicate that NAFLD and MS are not only related to the metabolism of lipids and carbohydrates; the occurrence of MS, T2DM, and NASH is also associated with uric acid, a natural waste product of purine metabolism. In a study of NAFLD patients, 53% of whom had NASH, the highest serum uric acid levels were found in the older participants and those with higher BMI, blood pressure, TG levels, and total cholesterol, and reduced HDL. In addition, those with the highest uric acid levels also had advanced liver steatosis [166]. On the other hand, fatty liver is not only related to purine metabolism but also associated with dysregulation of liver aminotransferase expression, which results from disturbances in amino acid metabolism [167].

In addition to dysfunctional uric acid or amino acid metabolisms, NAFLD and NASH patients often demonstrate changes in iron metabolism. NASH patients demonstrate elevated levels of ferritin and transferrin, which are responsible for the storage and transport of iron in the organism [168]. Increased ferritin levels are also noticeable in half of NAFLD patients [8]. Interestingly, hyperferritinemia is a predictor of advanced liver fibrosis and an increased risk of death [169]. Disturbances in iron metabolism may contribute to the oxidative stress and IR present in MS and NAFLD. Excess iron enhances the Fenton reaction, which converts hydrogen peroxide to hydroxyl radicals. Moreover, mice with higher dietary iron intake demonstrate increased levels of 15-lipoxygenase. Normally, it is associated with the physiological leakage of the peroxisomal membrane. Nevertheless, with increased iron concentration, this enzyme is more active, resulting in elevated levels of hydrogen peroxide, thus in lipid peroxidation [170]. This consequently leads to the production of MDA, which activates stellate cells in the liver and fibrogenesis [171]. In rats, a carbonyl-iron diet leads to the activation of NF-κB and a reduction in antioxidant capacity [172]. Thus, rats fed a diet depleted of iron exhibit lower oxidative stress due to decreased production of free radicals in the liver and reduced lipid peroxidation [173]. 

Recent studies indicate that the development of NAFLD is influenced by the gut, specifically the gut microbiota, which are associated with obesity and IR [174]. Different microbiota is observed in the feces of obese and lean people. For instance, obese people and mice have reduced levels of Bacteroidetes, i.e., the main phylum of beneficial bacteria indirectly involved in various metabolic pathways [175]. Men suffering from MS and IR who received intestinal microbiota infusions from non-obese people demonstrate improved insulin sensitivity [176], and the subjects with MS demonstrated higher numbers of bacteria that produced butyrate after treatment [176]. Oral administration of butyrate to mice reduces IR, while sodium butyrate administered to rats protects against NAFLD, even when fed with a high-fat diet [177,178].

#### 3.1.1. Obesity

As it was mentioned, obesity is a major contributor to the development of MS but is also strongly associated with the prevalence of NAFLD. Therefore, it can be assumed that this is the common denominator between these two diseases [179]. Nevertheless, despite the presence of fatty livers also in lean individuals, IR is much more related to higher fat accumulation than lower fat body content. The relationship between obesity, MS, and NAFLD is noticeable because each disease derives mainly from a sedentary lifestyle and a high-fat and high-carbohydrate diet [179]. 

Over 95% of morbidly obese patients undergoing bariatric surgery are diagnosed with NAFLD [180]. Up to 12% of these people have severe fibrosis or cirrhosis [2]. Interestingly, simple fatty liver disease only converts into NASH in 3% of lean patients, but in 20% of obese patients [18]. As the prevalence of obesity increases, so does the frequency of MS. A multi-cohort study of more than 160,000 obese Europeans found that MS occurred in 43%–78% of men and 24%–65% of women [181]. Although MS and obesity are linked with NAFLD, even 29% of patients with fatty liver are non-obese [182]. Moreover, obesity and NAFLD, including fibrogenesis and cirrhosis, occur in children as often as in adults [18]. 

Comprehensively, it has been confirmed that the degree of steatosis correlates positively with BMI [183,184]. However, while these patients appear to have normal BMI, they show lower fat content in the legs but higher fat accumulation in the midsection, as abdominal obesity; a similar phenomenon is encountered in MS [185]. Lipodystrophy, the non-uniform distribution of body fats, is often present at healthy BMI; it is believed to be important in the mechanism of MS and is a risk factor in NAFLD [186,187]. In general, lipodystrophy, obesity, NAFLD, and MS demonstrate disturbances in visceral fat tissue, which together with subcutaneous tissue, form white adipose tissue [188]. Often, the visceral adipose tissue/subcutaneous adipose tissue ratio is increased in NAFLD [189]. Excessive production of FFAs by visceral fat tissue in a pathological situation impairs the PI3K/Akt signaling pathway, increases oxidative stress, and reduces insulin sensitivity [5].

Obviously, since adipose tissue influences inflammatory pathways through the production of hormones, adipokines, cytokines, and chemokines [190], obesity is considered a condition of low-grade chronic inflammation [18]. The cytokines secreted by adipose tissue have not only proinflammatory properties but also produce protective proteins, such as IL10 and adiponectin [191]. TNF-α is the first cytokine to link obesity with IR [192]. Low levels of TNF-α are secreted physiologically by adipose tissue, but its expression was found to be increased in obese rodents [193], and its mRNA levels are also elevated in obese IR patients [194]. Moreover, obese mice with depletion of TNF-α demonstrated significantly improved insulin sensitivity compared to WT mice [195]. 

In contrast, IL6, a proinflammatory cytokine, can prevent damage to the liver. It is believed to protect the liver against hepatic fibrosis by attenuating oxidative stress and mitochondrial dysfunction and enhancing hepatocyte proliferation [190]. Although inhibition of IL6 activity triggered IR in mice, studies using both animal models and NAFLD patients have shown that the severity of disease correlates with IL6 level [196]. Indeed, although IL6 protects against damage, it may also cause steatosis [197]. In addition, the elevated levels of TNF-α and IL6 observed in obese patients activate JNK signaling and IR [10]. Besides producing the cytokines, adipose tissue also secretes leptin, which stops hunger. In animal models of NAFLD, a higher level of leptin activates fibrogenesis, and in humans with this disease, its rise contributes to an increase in the severity of steatosis [198,199,200]. Although obesity is not required to diagnose NAFLD, studies show that it is a serious risk factor of fatty liver disease.

#### 3.1.2. T2DM

Diabetes mellitus can be classified into type 1 diabetes (T1DM), i.e., non-insulin-dependent type, IR-related insulin-dependent T2DM (most occurring cases of diabetes), and other less common diabetes [201]. T2DM is a multifactorial disease in which the organism is unable to respond appropriately to insulin concentrations due to abundant nutrition and ensuing obesity [202]. It belongs to the diagnostic criteria cluster for MS and, along with obesity and IR, is very closely related to NAFLD [203]. Most patients with both NAFLD and T2DM fulfill all criteria for MS, so it can be assumed that the diseases are dependent on each other [187]. NAFLD patients suffering from T2DM concurrently have a much more severe disease course than non-diabetic patients [204].

More than 70% of T2DM patients also suffer from NAFLD [179,205,206]. Among the hospitalized ones with T2DM, 45%–75% also had fatty livers, and according to the population study, 30%–70% of diabetics had NAFLD [207]. In a meta-analysis examining 35,599 cases of T2DM, 59.67% of cases had NAFLD, while 77.87% were simultaneously obese with fatty livers [208]. In another meta-analysis of 49,419 people with T2DM, 55.5% of them had NAFLD [22]. In general, advanced liver fibrosis is a common occurrence in patients with T2DM [209]. Studies with magnetic resonance imaging, magnetic resonance elastography, FibroScan, and biopsy were used to evaluate the steatosis and stiffness of the liver; the results indicate that 37%–70% of T2DM patients had NASH and 7%–50% had advanced fibrosis [207,209,210,211,212]. While NAFLD can be observed in the occurrence of T2DM, the opposite is also true: T2DM or dysregulation of fasting glucose is observed in 18%–33% of NAFLD patients [213]. In a study of 88 NAFLD patients, 69 had T2DM or impaired glucose tolerance, and patients with advanced fibrosis showed a higher IR [214].

While T2DM often precedes the onset of NAFLD, NAFLD is also considered a predictor of T2DM [215]. Based on the research, it is not possible to conclude unambiguously whether NAFLD is the cause or effect of T2DM since NAFLD increases the risk of diabetes and vice versa [216]. It is estimated that diabetics are 5.36 times more likely to develop NAFLD than healthy people [217]. The results of numerous biopsies have shown that T2DM is a predictor of NASH and advanced fibrosis [218]. A study of 108 double biopsies with a 6.6-year interval found that the group of people who demonstrated deteriorated fibrosis after this time had a higher percentage of diabetes than those without progression of fibrosis [219]. T2DM also increases the risk of cirrhosis, liver failure, and hepatocellular carcinoma, as well as hospitalization and death, as a consequence of NAFLD [218]. Other data show NAFLD to be a significant risk factor in the development of T2DM [220,221,222]. IR almost always accompanies NAFLD, as it facilitates the course of diabetes [205]. The presence of above 10% of hepatic liver fat content significantly increases systemic IR and the risk of T2DM [223]. The chance also rises in the case of the co-occurrence of NAFLD and MS [215]. Moreover, the presence of the *PNPLA3* I148M variant, related to NAFLD, elevates the risk of T2DM and IR [224,225].

Visceral adipose tissue plays an important role in the development of T2DM, as well as NAFLD, by producing cytokines and activating the NF-κB pathway [226,227]. Increased gluconeogenesis and attenuation of insulin sensitivity are observed due to the activity of protein kinase Cε, fetuin A/B, retinol-binding protein 4, or selenoprotein P. T2DM patients typically demonstrate higher hepatic fat content and inflammation. In addition, lipogenesis is continuously active due to IR, and dyslipidemia occurs. Thus, patients demonstrate elevated blood pressure, and low-density lipoprotein (LDL) and TG levels, as well as decreased HDL. T2DM contributes to an increased influx of FFA into the liver, which induces the onset and progression of NAFLD. Obesity is exacerbated, and the disease worsens in response to oxidative stress, ER stress, lipotoxicity, and the activity of proinflammatory cytokines released by Kupffer cells and adipocytes, leading to apoptosis or necrosis [202,228,229].

T2DM contributes to the dysregulation of metabolic and inflammatory pathways. Furthermore, cases with both T2DM and NAFLD demonstrate more severe hyperinsulinemia, dyslipidemia, and hepatic IR than those with T2DM alone [221,230]. Higher levels of ALT and GGTP indicate an increased risk of T2DM. However, even NAFLD patients with the proper ATL serum levels, but with T2DM and IR, have a greater chance of higher grade steatosis and a more severe course of the disease [205]. Studies show that in patients with T2DM, a high-fat diet rich in pathogen-associated molecular patterns and altering gut microbiota may stimulate TLR2 and TLR4 and lead to elevated intestinal permeability and inflammation. T2DM is also related to mitochondrial dysfunction since reduced ATP production levels have been detected in diabetics after fasting or fructose administration [231,232]. To summarize, T2DM is closely associated with the development and progression of NAFLD.

### 3.2. The Role of BER in NAFLD

Due to the fact that oxidative stress plays a considerable role in NAFLD development and progression, the mechanisms that are responsible for the repair of oxidative injure must function efficiently enough to handle the problem. Free radicals and mitochondrial dysfunction can promote damage to genetic material. Therefore, it is important that DNA repair pathways are not impaired and that lesions are eliminated effectively.

#### 3.2.1. BER and Adipose Tissue Metabolism

Mitochondria play an important role in the pathogenesis of NAFLD and are closely associated with IR through FFA oxidation and lipotoxicity [233]. The huge impact of mitochondria is exemplified by the relationship between NAFLD and MS, both of which are linked to mitochondrial dysfunction and oxidative stress [234,235]. Mitochondrial dysfunction and the intracellular accumulation of diacylglycerol and ceramide disrupt insulin signaling pathways. Furthermore, a significant role is played by the relationship between IR and ROS generated as by-products of the energy metabolism in the mitochondria. The increased supply of nutrients and the catabolic reactions present in obesity, NAFLD, or T2DM, increases the number of electrons provided to the ETC, thus elevating the proton gradient across the inner mitochondrial membrane. Instead of increasing the synthesis of ATP, this results in overproduction of ROS, which not only induces oxidative damage to the DNA, proteins, and lipids, but also act as signaling molecules modulating insulin signaling [236]. 

In addition to the damage to nuclear DNA, IR also causes damage to mtDNA. The most important DNA repair mechanism in mitochondria is BER, which is able to eliminate oxidative lesions like 8-oxo-dG adducts from mtDNA, as in its nuclear counterpart [237]. BER recognizes damage as oxidative, alkylation, deamination, and AP sites. The process consists of four steps: (i) recognition, (ii) excision, (iii) synthesis, and (iv) ligation of DNA. The recognition and removal of lesions are performed by glycosylases, including oxoguanine glycosylase (OGG1), nei-like DNA glycosylase 1 (NEIL1), or MutY DNA glycosylase (MUTYH). The prepared structures are then processed by apurinic/apyrimidinic endodeoxyribonuclease 1 (APEX1), polynucleotide kinase 3’-phosphatase (PNKP) or 5’-deoxyribose phosphate (dRP)/AP lyase, respectively, which are able to cleave the phosphodiester bonds [238]. The synthesis stage is performed by DNA polymerase, which inserts correct nucleotides or nucleotides in the generated gap. Single nucleotides are repaired by short patch BER (SP-BER), while groups are replaced by long patch BER (LP-BER) in which a flap structure-specific endonuclease 1 (FEN1) acts to create and remove 5 ‘overhanging flaps [238]. In the final step of BER, DNA strands are sealed by DNA ligase 1 (LIG1) or 3 (LIG3), along with interacting proteins Poly(ADP-ribose) polymerase 1 (PARP1) and X-ray repair cross-complementing protein 1 (XRCC1) [239,240]. 

**Figure 3:**
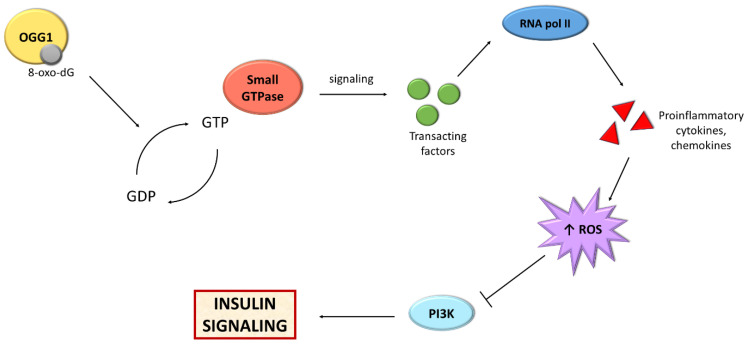
Scheme presenting the relationship between BER and insulin signaling. Abbreviations: GDP: guanosine diphosphate; GTP: guanosine triphosphate; OGG1: oxoguanine glycosylase; PI3K: phosphoinositide 3-kinase; RNA pol II: RNA polymerase II; ROS; reactive oxygen species.

Recent studies highlight the importance of one of the BER mechanism components, i.e., OGG1, in the metabolism of adipose tissue (Figure 3). Findings show that mice with knockout of the *OGG1* gene (*OGG1^−/−^*) demonstrated an increase in weight and fat content with age [241]. After 10 weeks of high-fat diet feeding, mice were heavier in comparison to WT, and their livers were fatty. These mice demonstrated hyperinsulinemia and impaired glucose tolerance, which results in IR and a prediabetic state. Moreover, these animals showed a higher respiratory exchange ratio, which suggested a prevalence of carbohydrate metabolism over fatty acid oxidation. In addition, the expression of genes associated with hepatic fatty acid oxidation, the amount of glycogen in the liver as well as serum fasting ketone levels were reduced, suggesting a decrease in liver lipid oxidation [241].

Furthermore, *OGG ^−/−^* mice show an increase in skeletal muscle lipid accumulation and dysregulation of genes associated with lipid uptake and mitochondrial fission in muscles. Muscle functionality, i.e., grip strength and treadmill endurance also decreased [242]. The OGG1-deficient mice also demonstrated hyperglycemia, higher mtDNA 8-oxo-dG levels, and elevated liver glycogen concentration due to the inability of suppression of gluconeogenesis in the fed state. The ability of ECT was reduced, which strengthens DNA repair as an important factor in the regulation of metabolism [243]. 

The OGG1 enzyme exists as nuclear and mitochondrial variants. It has been shown that the lack of a nuclear variant is compensated by the action of the mitochondrial variant. *OGG1 ^−/−^* mice with mitochondrial-targeted OGG1 show consistent mitochondrial expression of this gene. These animals are protected against obesity, hepatic lipid accumulation, IR, and adipose tissue inflammation [244]. Komakula et al. [245] demonstrated the importance of OGG1 in lipid accumulation and an increase in adipocyte differentiation. The lack of the gene increases fat content and adipogenesis, while overexpression attenuates them. These studies confirm the importance of DNA repair in fat metabolism. Moreover, the presence of the c.1245C>G (substitution of cysteine for serine at codon 326, Ser326Cys; rs1052133) polymorphism in the *OGG1* gene increases the risk of cancer, neurodegenerative, cardiovascular, and metabolic diseases, including T2DM and MS [246]. 

In addition, some studies indicate that mice lacking another glycosylase, NEIL1, show metabolic alterations. Animals fed a high-fat diet showed obesity and glucose intolerance present in MS [247,248]. The findings confirm in general importance of BER pathway in NAFLD (Table 1).

#### 3.2.2. DNA Repair in NAFLD

The above studies showed mainly the influence of BER-related proteins on adipose tissue and lipid metabolism, which in turn influence the development of IR. In contrast, the following research provides a general picture of the relationship between NAFLD and alterations in DNA repair pathways, mainly in BER.

Recent studies have examined the relationship between the expression level of genes involved in BER and fatty liver. MCD diet-fed mice were found to demonstrate increased gene expression of thymine-DNA glycosylase and APEX1, enzymes related to BER [252]. In addition, expression is also influenced by pioglitazone, a drug used in T2DM, which can improve insulin sensitivity and the general condition of NASH patients. When administered to animals, pioglitazone reversed high-fat diet-induced steatosis and increased the expression of the *OGG1* and *MUTYH* genes. This suggests that DNA repair may have a significant impact on improving fatty liver [250]. Although fatty liver in animals is observed to co-occur with elevated DNA repair activity, Gao et al. [249] report that *MUTYH* expression was decreased in animals fed MCD diet. In the case of rats fed a fructose-rich diet rich, a decrease in the expression level of gamma DNA polymerase and a lower mtDNA copy number could be observed [253]. 

NAFLD is associated not only with BER but also with other DNA repair mechanisms. The liver steatosis appears to be associated with *Gadd45α*, which encodes the protein involved in cells differentiation, apoptosis, necrosis, and base and nucleotide excision DNA repair. *Gadd45α* is upregulated in NAFLD patients, probably in response to oxidative and ER stress in hepatocytes. In addition, it plays a protective role against steatohepatitis in *Gadd45α^−/−^* mice fed MCD diet. In comparison to a group of WT animals, more severe hepatic inflammation and fibrosis have been observed [254,255].

In patients with obesity and fatty livers, the activity of the nucleotide excision repair, pathway responsible for the repair of lesions caused by UV light, environmental factors, or some DNA adducts, has been significantly decreased [251]. These findings suggest that DNA repair mechanisms may be involved in the pathophysiology of NAFLD.

## 4. Conclusions

Despite the fact that NAFLD is a common disorder, many of the underlying mechanisms of pathophysiology still remain elusive. Nevertheless, it is clear that the key causative factors of the disease include fat accumulation, hepatic inflammation, oxidative and ER stress, as well as mitochondrial dysfunction. It is a multi-factorial disease that is closely associated with MS, T2DM, and obesity, and the common denominator of these conditions is IR. DNA repair mechanisms are also related to IR and the development of NAFLD; this is particularly the case for BER, which contributes to the course of the disease through OGG1. Nevertheless, further research is needed to better understand this topic.

## Figures and Tables

**Table 1:** The studies demonstrating the role of DNA repair in the course of lipid and insulin metabolism as well as fatty liver.

Research Characteristics (Studied Groups, Diet)	Main Outcomes	Paper
12-week old male ob/ob and WT (C57BL/6) mice. WT animals were fed: HFD with ethanol, HFD with dextrin maltose, MCD diet, or control liquid diet. ob/ob mice were fed: HFD (*n* = 6 in each cohort)	Mice fed MCD diet had: decreased *MUTYH* expression	Gao et al. 2004 [249]
Male and female *Neil1^−/−^, Neil1^+/−^* and WT (C57BL/6) mice	*Neil1^−/−^* and *Neil1^+/−^* mice had: severe obesity, dyslipidemia, hyperinsulinemia, and fatty liver	Vartanian et al. 2006 [236]
8-weeks old male C57BL/6 mice fed chow, HFD, or HFD with pioglitazone 100 mg/kg/day for 8 weeks (*n* = 5 in each cohort)	Mice fed HFD had: hepatic steatosis, improved by pioglitazone; increased malondialdehyde concentration and 8-oxo-dG, attenuated by pioglitazone; decreased gene expression of *OGG1* and *MUTYH*, reversed by pioglitazone	Hsiao et al. 2008 [250]
Male and female *Neil1^+/+^* and *Neil1^−/−^* mice fed chow and HFD (*n* = 6–10 in each cohort)	*Neil1^−/−^* mice fed HFD had: increased body weight, higher fat accumulation, moderate to severe hepatic steatosis, upregulation of hepatic expression of inflammatory genes, glucose intolerance	Sampath et al. 2011 [235]
Livers of 35 severely obese male and female patients with steatosis without inflammation or with NASH (each group divided into low or high MPO activity) (*n* = 8–9 in each cohort)	Patients with high expression of MPO had: reduced damage recognition capacity, decreased NER capacity	Schults et al. 2012 [251]
12-week old male *Ogg1^−/−^* (*n* = 6), *Ogg1^+/−^* (*n* = 6) and WT (*n*-= 6) mice (C57BL/6J), fed 10 weeks chow or HFD (*n* = 6 in each cohort)	*Ogg1^−/−^* mice fed HFD had: increased adiposity and hepatic steatosis, higher plasma insulin level, impaired glucose tolerance, higher respiratory exchange ratio, dysregulation of fatty acid oxidation and TCA metabolism genes expression, reduced hepatic glycogen stores, reduced fasting plasma ketones	Sampath et al. 2012 [241]
6-week old male C57BL/6J mice fed chow or MCD diet for 1 week (*n* = 6 in each cohort)	MCD diet-fed mice had: hepatic steatosis; higher gene expression level of thymine-DNA glycosylase and APEX1	Takumi et al. 2015 [252]
About 100-day old male Sprague-Dawley rats fed chow or a fructose-rich diet (*n* = 4–9 in each cohort)	Mice fed fructose-rich diet had: decreased gene expression of gamma DNA polymerase, reduced mtDNA copy number	Cioffi et al. 2017 [253]
12-week old male *Ogg1^−/−^* (*n* = 6) mice and WT (*n* = 6) (C57BL/6J), fed 10 weeks chow diet (*n* = 6 in each cohort)	*Ogg1^−/−^* mice had: dysregulation of expression of genes involved in mitochondrial fission, fatty acids oxidation, fatty acid uptake, TCA and pyruvate metabolism; increased muscle lipid content; decreased grip strength and treadmill endurance	Vartanian et al. 2017 [242]
Male and female *Ogg1^−/−^* and WT mice (C57BL/6N) fed or subjected to fasting for 24 h (*n* = 6–8 in each cohort)	*Ogg1^−/−^* mice in the fed state had: hyperglycemia, elevated insulin levels, higher liver glycogen content, increased accumulation of 8oxoG in mtDNA, reduced ETC capacity, decreased activity of PDH	Scheffler et al. 2018 [243]
Age-matched male *Ogg1^Tg^* and WT mice (C57BL/6 J) fed *ad libitum* chow or HFD for 12 weeks (*n* = 6 in each cohort)	*Ogg1^Tg^* mice fed HFD had: protection from diet-induced obesity, IR, and inflammation of adipose tissue	Komakula et al. 2018 [244]
Preadipocytes from 4–12 weeks old male WT (C57BL/6J), *Ogg1^Tg^* and *Ogg1^−/−^* mice fed chow diet (*n* = 5–6 in each cohort)	Preadipocytes from *Ogg1^−/−^* mice were more differentiated and accumulated more lipids than WT mice; from *Ogg1^Tg^* had reduced differentiation and lipid content	Komakula et al. 2021 [245]

Abbreviations: CPT1: carnitine palmitoyl transferase-1; ETC: electron transport chain; HFD: high-fat diet; IR: insulin resistance; MCD:methionine-choline-deficient; MPO: myeloperoxidase; mtDNA: mitochondrial DNA; ob/ob: genetically obese; PDH: pyruvate dehydrogenase; Pgc-1α: peroxisome proliferator-activated receptor gamma coactivator 1-alpha; TCA: tricarboxylic acid cycle; WT: wild-type.
